# A qualitative assessment of stakeholder perceptions and socio-cultural influences on the acceptability of harm reduction programs in Tijuana, Mexico

**DOI:** 10.1186/1477-7517-5-36

**Published:** 2008-11-20

**Authors:** Morgan M Philbin, Remedios Lozada, María Luisa Zúñiga, Andrea Mantsios, Patricia Case, Carlos Magis-Rodriguez, Carl A Latkin, Steffanie A Strathdee

**Affiliations:** 1 Division of International Health, School of Medicine, University of California San Diego, La Jolla, California, USA; 2 Pro-COMUSIDA, Tijuana, Mexico; 3 The Fenway Institute, Fenway Community Health, Boston, MA, USA; 4 Centro Nacional Para la Prevención del VIH/SIDA (CENSIDA), Ministry of Health, Mexico; 5 The Johns Hopkins Bloomberg School of Public Health, Baltimore, MD, USA

## Abstract

**Background:**

The Mexico-U.S. border region is experiencing rising rates of blood-borne infections among injection drug users (IDUs), emphasizing the need for harm reduction interventions.

**Methods:**

We assessed the religious and cultural factors affecting the acceptability and feasibility of three harm reduction interventions – Needle exchange programs (NEPs), syringe vending machines, and safer injection facilities (SIFs) – in Tijuana, Mexico. In-depth qualitative interviews were conducted with 40 community stakeholders to explore cultural and societal-related themes.

**Results:**

Themes that emerged included Tijuana's location as a border city, family values, and culture as a mediator of social stigma and empathy towards IDUs. Perception of low levels of both awareness and socio-cultural readiness for harm reduction interventions was noted. Religious culture emerged as a theme, highlighting the important role religious leaders play in determining community responses to harm reduction and rehabilitation strategies for IDUs. The influence of religious culture on stakeholders' opinions concerning harm reduction interventions was evidenced by discussions of family and social values, stigma, and resulting policies.

**Conclusion:**

Religion and politics were described as both a perceived benefit and deterrent, highlighting the need to further explore the overall influences of culture on the acceptability and implementation of harm reduction programs for drug users.

## Introduction

Tijuana's rate of illegal drug use is the highest in Mexico, with 14.7% of the city's population reporting a lifetime prevalence of ever having used an illegal drug (including marijuana), three times that of the national average (5.3%) [1]. Tijuana is situated on a major international drug trafficking route, and Mexico is one of the most important producers of heroin and methamphetamine entering the United States [2]. Due in part to its location on major routes for drug trafficking and migration, Tijuana has one of Mexico's fastest growing injection drug using (IDU) populations [3,4]. In 2003, there were an estimated 6,000 active IDUs and 200 shooting galleries in Tijuana, although the actual number of IDUs is likely much larger [5]. While syringes can legally be purchased in pharmacies in Tijuana, IDUs often report being refused or charged exorbitant prices [5]. Reduced HIV transmission among IDUs has been linked to access to needle exchange programs (NEPs) [6-8].

In this study, we asked respondents about the feasibility and acceptability of three harm reduction interventions including 1) NEPs, 2) syringe vending machines, and 3) safer injection facilities (SIFs). The structure and implementation of these programs differ markedly, but each intervention aims to decrease the circulation of contaminated injection equipment and transmission of blood-borne infections [7,9]. Beyond the provision of sterile syringes, both NEPs and SIFs provide the opportunity for integrated care, educational services, syringe disposal, and referrals for drug treatment, medical care and HIV testing [10,11]. These three interventions have been evaluated extensively and found to be effective in preventing the transmission of HIV and other blood borne pathogens without promoting or increasing levels of drug use, discarded syringes, or crime [12,6-8].

Although Mexico's federal Ministry of Health has published a document supporting NEPs, there appear to be small-scale programs operating in only six states – Baja California, Coahuila, Nuevo Leon, Oaxaca, Sinaloa, Zacatecas – with the most active being led by non-governmental organizations (NGO) in Ciudad Juarez and Tijuana [13]. At the time of writing, there were no known syringe vending machines or SIFs operating in Mexico.

Numerous articles discuss the empirical evidence for harm reduction interventions, but few describe barriers encountered prior to their approval [14,15]. For countries lacking a social and cultural environment amenable to harm reduction, there is a dearth of literature describing methods for facilitating the implementation of such interventions. Furthermore, few studies describe ways in which community stakeholders describe and define the problem of drug abuse, and how these views potentially affect their endorsement of harm reduction interventions. Previously, we described levels of acceptability and feasibility for implementing NEPs, syringe vending machines, and SIFs and factors that may influence their implementation in Tijuana, Mexico [16]. Herein, we specifically explored religious and cultural factors affecting the acceptability and feasibility of these harm reduction interventions in Tijuana, in an effort to inform the future development of culturally appropriate interventions in Mexico and potentially other countries.

## Methods

Between August 2006 and March 2007, trained Mexican and American interviewers recruited 40 key stakeholders who had direct or indirect interaction with IDUs in Tijuana, Mexico. In order to create a more complete understanding of attitudes toward these interventions, we used sampling methods adapted from the Rapid Policy Assessment and Response (RPAR) approach. The RPAR method, as operationalized by Lazzarini and colleagues, [17] combines traditional legal analysis with empirical data collection to assess how structural factors can impact community-level health interventions. This mixed methods approach, which integrates qualitative data on implementation of laws, policies and practices with locally important policy questions was recently used in four countries (Poland Russia, Ukraine, and Kazakhstan) and found to be useful in identifying policy issues and guiding interventions [18-21].

We adapted RPAR sampling methods by constructing a targeted sampling grid and interviewed local stakeholders at two levels (system and interactor) in order to obtain diverse perspectives. These stakeholders included interviewees from five sectors; health, religion, legal, pharmacy, and rehabilitation. Systems level stakeholders were chosen because they possess oversight of critical components within a given system and included respondents in each of the five sectors. Interactor level informants operate in sectors that affect IDUs' attitudes, behaviors, and access to syringes, and typically have daily contact with IDUs. Interactors provide practical on-the-ground information about the implementation of drug policies and the limits of risk reduction interventions and offer a unique perspective because of their understanding derived from interacting both with IDUs and policy makers.

The targeted sample was constructed after a master list was created of all Tijuana stakeholders who were involved with drug use policy, health policy, or program implementation at the systems or interactor level in each of the five sectors. From this list, key informants were chosen based on their level of experience, time spent in Tijuana, and willingness to be interviewed. Specific informants – politicians, judges, pharmacy owners and clerks, pastors, methadone clinic doctors, ministry of health officials, and directors of drug treatment programs – were interviewed based on their understanding of, and ability to affect change in, the drug injecting risk environment. Participants were not reimbursed for their participation in interviews.

After being recruited for the study and providing voluntary and informed written consent, each participant was asked 10 quantitative questions to assess socio-demographic information such as age, gender, and education level. The interviews were semi-structured and provided opportunities for the interviewers to probe further into topics about which the interviewee had particular expertise or opinions. The topic guide allowed flexibility to focus on specific interventions (i.e. NEPs, syringe vending machines, SIFs), social-cultural barriers and facilitators of implementation, and suggestions for future programs. Prior to intervention-specific questions, definitions of key interview terms were given to each participant to promote respondent understanding of interview terminology. Specific questions included "Which harm reduction interventions do you see as feasible in Tijuana's current socio-political context?" "What are possible cultural and social obstacles to implementation?" And, "How does Tijuana's location as a border city affect its drug culture?" Interviews were conducted in private locations including homes, offices, or places of work. This study was approved by Institutional Review Boards at University of California at San Diego and Tijuana General Hospital.

Interviews were approximately one hour long, conducted in Spanish, and digitally recorded. The audio files of the interviews and transcripts were anonymous, and identified only by code numbers. Audio files were destroyed after transcription and translation. Native Spanish speakers conducted verbatim transcription and translation of the in-depth interviews. Translations were validated by two bilingual individuals. A "do not translate" list including street jargon and slang words was created, along with a corresponding glossary, in order to preserve the connotations and meaning of the original Spanish-language version.

Content analysis was conducted concurrently with data collection to allow revision of the interview guide to reflect new information. The analyses focused on generating themes such as acceptance of harm reduction in the Mexican context, cultural and political barriers to implementation, and the socio-cultural feasibly of, and suggestions for, the implementation of harm reduction interventions. Transcripts were first hand-coded by two investigators who, after reading a cross-section of the interviews, created a preliminary codebook containing key concepts and categories. The investigators then applied these codes to ten interviews in order to modify and create more nuanced versions of the codes. Using qualitative data analysis software, ATLAS.ti [22], interviews were uploaded and coded by two members of the study team using the preliminary codes. Any discrepancies between coders were discussed among the investigators and resolved.

## Results

A total of 40 stakeholders were interviewed from the following sectors: health (n = 13), rehabilitation (n = 8), legal (n = 11), pharmacy (n = 3), and religious (n = 5). Well over half of respondents were male (67%), with a median age of 42 years (range 31–71 years). When asked about their political orientation, 28% responded liberal, 52% moderate, and 20% conservative. None described themselves as 'very liberal' or 'very conservative'.

Of the three interventions, NEPs were seen as the most acceptable with 75% supporting, followed by vending machines (65%) and SIFs (58%). Levels of perceived feasibility were much lower than acceptance; half of participants (53%) believing the implementation of NEPs to be possible, followed by 38% for vending machines, and 25% for SIFs. The analyzed themes, response and context of harm reduction, religious barriers, political barriers, and suggestions for implementation are described below.

Interviewees consistently described Tijuana as a city with a unique mix of cultural, geographic, and social factors that contribute to high levels of drug use; factors included a large transient population, high numbers of deported individuals, and a physical location along a drug trafficking route. One health sector interviewee said:

Tijuana is a city with a large floating population, where people often come with the intention of crossing into the United States. And when they cannot or do not, many of them remain anchored here in the city, without family, without a place to live, they start loitering in public; then they make contact with people who have these problems, and they often go so far as to acquire the illness or the problem (Male, 45, Health Sector).

### Socio-political Context of Harm Reduction Intervention

Levels of acceptance and support for harm reduction interventions differed by interview sector; those in the health sector expressed the most support, the religious sector the least. The majority of individuals, however, accepted at least one form of the three harm reduction interventions. Investigators observed a dichotomy within respondents themselves: individuals who personally supported harm reduction interventions, yet did not see them as feasible within Mexico's current socio-political context. As a female in the health sector explains,

Because of the beliefs within our culture, it wouldn't be practical. Maybe in other countries, but not here. That said, I think it would be very practical because the person, the drug user wouldn't have a problem and they can go at whichever moment is convenient for them ... it would be very good, but the reality is that I don't see it as likely to be implemented (Female, 42, Health Sector).

Along with this individual in the health sector, a legal sector respondent didn't feel that Mexico was prepared to accept harm reduction. Her rationale was that people in the current socio-political context were not open to such an idea, in comparison to more liberal countries, and thus would be prejudiced, in allowing these interventions.

Look, if the community was prepared intellectually and culturally and if we didn't have so much prejudice then the programs would work, but [unfortunately] we are not prepared. First of all, we need people to work on this law, and need to modify it because we need to have a law that support such programs. Personally, I like the idea ... but we are still not ready for this (Female, 42, Legal Sector).

Another aspect of socio-political environment that was discussed as a potential barrier was the political system and its lack of openness to harm reduction. As one individual described,

I see it as something difficult to implement because of the same; the culture. And it is not just in Tijuana, I guess I see it as a bit too difficult because of politics and for the government to be open to such establishments, and the society, too. There would be a struggle to open such establishments, little by little with time it could be implemented ... and would be accepted by the society (Female, 35, Legal Sector).

Respondents representing religious sectors also mentioned that the government was not interested in providing support for harm reduction programs.

I think the government is not interested because it feels that there is no solution for these people, they are not interested that many people have a drug problem ... if you watch the news you are not going to hear about a program concerned with drug users or alcoholics. No, you don't hear this and I think it is because the government is not interested in these types of people (Female, 55, Religious Sector).

Many participants felt that the cultural context of Tijuana was not amenable to the harm reduction interventions proposed in the interview. One respondent alluded to their perception that :

The culture that we have is the barrier, and maybe the principal barrier, because the political decisions are derived from it, as well as personal actions. We have these concepts in our culture that drug users continue to be delinquents, and these then become impairments because it affects politics on various levels (Female, 42, Health Sector).

Throughout the discussion of the role of culture in the acceptance and feasibility of harm reduction, the most salient factors to emerge concerned the influence of religion and politics on the rules and norms of Mexican culture. These are further described below.

### Religion as a Barrier to and Facilitator of Harm Reduction

We interviewed individuals from both the Catholic and Evangelical Churches, though the majority of people referred simply to "The Church," and not a specific denomination in their interview. When the topic of religion was discussed by informants, there was a distinct divide between those involved in the religious sector and those outside of it. Those who did not represent a particular religion (or were not identified as a representative of a particular religion) named The Church as a barrier to these harm reduction interventions; interviewees who represented specific religious denominations appeared to see themselves as providing services superior to standard harm reduction interventions. As an individual in the rehabilitation field described:

I think we all agree that there is delinquency, that Tijuana as a society has a problem with both the circulation and distribution of drugs ... but what about the church? Many times I feel that they are in opposition to this type of program because they are not yet aware of the problems that are outside the church (Male, 35, Rehabilitation Sector).

In contrast, those involved with The Church saw themselves as nurturers providing a much-needed service. A member of the religious sector described his role as follows,

Well, the church sees these people as precious humans, we see the potential that they have that they don't seem to know ... They come and they recover and they reenter society as different people, as people that are valued by society, their decisions are accepted by society and their actions are believable, they endorse what they speak because they have been prepared, and they have been instructed in the word of God (Male, 35, Religious Sector).

Some individuals who represented a religious perspective only saw harm reduction as something dangerous or risky, while others noted its potential as a way to stem disease. An individual in the religious sector voiced that he would not support harm reduction interventions, while simultaneously acknowledging a lack of understanding of the problem as a whole.

It is like saying to a child here you have a gun and use it, and the risk is there that the child will shoot it or use it, it is very risky, dangerous, delicate, too much exposure. I will repeat that I feel that there is a lack of culture, preparation, even a consciousness on this matter, and that is why we haven't talked much about the problem, it is not known (Male, 46, Religious Sector).

In contrast, an individual in the religious sector voiced support for harm reduction, saying he had worked previously with drug users and understood the potential problems.

[I think harm reduction] is good, but people would say we are condoning drug use, or approving it, but I want to ask whether the persons who are helping the addicts not take drugs are any different? Sometimes one says that if I approve this intervention it means that I am approving taking drugs. It is not that (Male, 59, Religious Sector).

Some respondents suggested a practical approach to religious individuals who may impede the development of these or other health-related programs, by asking them simply not to act to stop programs. As one individual in the health sector suggested,

I think there are questions or health issues that do not concern the church, therefore, the church should not put any obstacles when it comes to the health or the lives of a whole community. We can respect their ideology, but ask them when it comes to health issues for them to respect the work that we are doing (Female, 50, Health Sector).

### Politics as a Barrier and Facilitator of Harm Reduction

Many respondents criticized the Mexican government for what they saw as a lack of initiative and willingness to provide programs for drug users. What they saw as the government's reason for lack of interest varied, however. One individual in the religious sector believed that,

The government is not interested because it feels that there is no solution for these people, they are not interested that many people have a drug problem ... The government doesn't seem to worry. If you watch the news you are not going to hear about a program that has concerns about drug users or alcoholics, do you understand? I think it is because the government is not interested in these types of people (Male, 46, Religious Sector).

In contrast, an individual in the pharmacy sector saw it as tied to corruption and lack of financial will, something he contrasted with the U.S. government.

I wish that the government [could do something], I believe that the American government can do something, in Mexico however many times there is corruption and many programs are not done because they just want to make money on these types of things and this is precisely what should not happen, but there are many corrupt officials (Male, 71, Pharmacy Sector).

The majority of individuals mentioned the government as a possible barrier to the implementation of harm reduction, or suggested that the political sector should be avoided. As one individual in the legal sector noted,

I think we need to fight for the social context only, and avoid the political context, because these are general topics that don't concern political parties, age, or sex. So then, it would please me if there was some political will among the politicians to forget color and support programs for the sake of all society (Male, 32, Legal Sector).

An individual in the health sector had a more optimistic approach about the possibility of working within the government to create harm reduction programs, though acknowledged it would not be easy. As she explained,

I think we need to work and show its necessity; independent of the ideology of the political parties or the administration that is governing here. It is not particularly easy right now because a very conservative party runs the government. As a result, we need to work in a very objective way, proving the necessity for public health, so they can independently support our ideology (Female, 50, Health Sector).

### Socio-Cultural Readiness and Suggestions

Along with numerous criticisms of current policies and barriers to implementing harm reduction, individuals discussed Tijuana's socio-cultural readiness for harm reduction and what could be done to facilitate its implementation. One individual in the rehabilitation sector identified a need for legislative change:

First of all there should be a law that addresses how these programs should be organized so it can be done from a legal framework. The addiction problems and the delinquency problems in the community should all be connected in order to bring these types of health problems together ... We have to make a lot of modifications in terms of what the law allows (Male, 44, Rehabilitation Sector).

A health official also suggested what he could do within his own job capacity to inform and increase awareness for those in decision making roles. He highlighted that it was a joint responsibility to provide politicians with the knowledge to make informed decisions,

[We need to] establish more clear politics to avoid confusion when it comes to decision makers, but if I don't provide them with a well written document at the time of their making decisions, then we are not going to be able to move forward ... I am convinced that we cannot do this alone. As a society we have to get informed, to read about it, and to know that these people are not isolated from the rest of us, that they are integrated with our society; we need to accept them and help them in some way (Male, 47, Health Sector).

Along with politicians, individuals from the other sectors stressed the importance of working within The Church, and integrating religious leaders into existing programs to help foster support.

As one individual in the rehabilitation sector described,

The most effective way would be the participation of everyone, to make them aware of the problem that we have, make public policies that contribute to family values, make a regulation or a law that controls the resources or the designation of resources to all the rehabilitation centers ... We need to do a campaign and find political alliances. We need an ally even in the Catholic Church to reduce the radicalism of these groups right? And society too, cause the government can't really do something without society's support, and the more society is involved the less the government the better things get done, so with better social organization of course (Male, 44, Rehabilitation Sector).

Though the Mexican government has begun to show small scale support, individuals stressed that a great deal still needs to be accomplished, and suggested ways of supporting the development and implementation of programs. One person responded with a series of specific suggestions, saying:

We can show the results of the studies that we have made so far, and show them that in Tijuana we are seeing behaviors very similar to other countries where the epidemic has had very serious complications. We can convince them by showing the cost effectiveness and benefit of these programs, that it is cheaper to promote or give information and give away syringes and condoms, than spend millions of dollars in treatment...As for the implementation strategy, we need to do this gradually, with well planned changes so it won't create resistance, because of the mentality of the government, or the mentality of the conservative party (Female, 50, Health Sector).

## Discussion

This research focused on societal level factors as previous research with IDUs has suggested that the transmission of blood borne infections is strongly shaped by socio-cultural norms, politics, and religion [23,9]. This qualitative study among key stakeholders who may be able to influence policy examined the barriers to, and acceptance of, harm reduction interventions – needle exchange programs, syringe vending machines, and safer injection facilities – in Tijuana, Mexico. Certain themes were repeatedly mentioned by different participants, suggesting that the data had reached saturation. Though the majority of respondents supported harm reduction, some sectors, including religion, were almost unanimously opposed. These findings indicated the important role socio-cultural context plays in determining the acceptance of harm reduction, including religious and political opposition. Individuals also outlined key suggestions – raising awareness, creating new laws, working with community leaders – to increase feasibility and thus promote the implementation of harm reduction interventions. One factor to emerge from this research was the differing questions of what the "problem" in in relation to injection drug use, and who should define this problem.

Many interviewees described Mexican culture – specifically discussed within the context of religion and politics – as a barrier. The research team observed that the term "culture" was applied in a variety of ways, including 'drug culture' and 'culture of acceptance of interventions'. Two distinct patterns emerged in the way individual's used the words "Mexican Culture." The first described harm reduction as something that would be successful in other countries that were "more developed," but not in Mexico itself. The second described culture as something that contained multiple factors that were still taboo to discuss (e.g., sex, drugs) and stressed a general lack of awareness among the general population. These issues alluded to the perception that it is currently "culturally unacceptable" for harm reduction to be implemented as it was seen as at odds with Mexican socio-cultural norms. This issue of harm reduction being contrary to a specific culture was also found in Russia. Tkatchenko-Schmidt et al [24] found that a key barrier to harm reduction scale-up was cultural unacceptability, and was related to two factors; the legacy of policies of the communist past and the involvement of international agencies in harm reduction programs [24].

Although we did not specifically ask about religion, this theme was repeatedly mentioned as both a barrier and facilitator. Religion was consistently mentioned as a barrier, and religious sector interviewees continuously repeated that harm reduction was not only insufficient, but that it would promote further drug use. One of the key factors in determining receptivity to harm reduction is how the problem of drug use is framed, which in turn affects what people see as the most reasonable approaches to solving the problem. For example, the majority of stakeholders saw the problem from a health standpoint, in that any intervention that would lower risk for diseases or drug related harm should be implemented; religious stakeholders saw drug use as something that must be stopped immediately. Many mentioned abstinence as the only acceptable option, a finding consistent with previous research that religious organizations associate harm reduction with what they deem risky and immoral behavior [25]. Our research is supported by other findings describing the integral role of religion in communities, and how critical the support of the church and clergy is to the success of government-sanctioned harm reduction programs [15,26,16,24,27]. More specifically, while researching the feasibility of NEPs, Vlahov et al (2001) found that leaders among African American Churches were particularly opposed [27]. The Catholic Church and Mexican culture are intricately intertwined – 88% of the population considers themselves Catholics – and individuals working in the health care field can find themselves divided between personal support for harm reduction and their religion's denunciation of such strategies [28].

A similar divide also occurred during the abortion debate in the early 1990s as Catholic bishops in the state of Chiapas threatened excommunication of lawmakers who may have approved a bill legalizing abortion [29]. In this case, many individuals felt the Catholic Church overstepped its influence, and the majority of Catholics reported feeling that a politician's personal religious beliefs should not affect their legislative decisions on health issues and that efforts should be focused on decreasing the Church's political influence [30]. Regardless of this assertion, it is difficult to avoid the Church's influence as it plays such a large role in Mexican culture [30].

Previous studies in other settings have examined how an individual's relationship to religious institutions, and perceived spiritual support, can reduce risk behaviors and is also an independent predictor of abstinence from illicit substances [31,32]. Research in Brazil found that various Christian religions interacted with drug use and rehabilitation in different ways; religions with an evangelical orientation were more likely to use religion as an exclusive form of treatment, even eschewing medical intervention and pharmaceuticals, while Catholics were less likely to reject a doctor's intervention [33]. Interviewees also reported that, along with religious faith, other factors that helped drug users remain drug free were the support and positive pressure provided by the program staff [33]. Though research has focused on the role religion can play in an individual's life, little research has been conducted examining the role a church or religious leader plays in determining acceptance of harm reduction [34,35].

Many interviewees noted that a lack of political will and government support served as a barrier to implementing harm reduction. As our research was conducted during an election year, it was not surprising that politicians were hesitant to openly support harm reduction. The importance of political support in creating a system amenable to harm reduction interventions has been noted in other locales, including Russia, Malaysia, Vietnam, and China [36-38,24]. Bluthenthal et al [39] found a 46% increase in the total number of California's NEPs after the passage of an assembly bill eliminating criminal prosecution for the distributions of syringes. Likewise, after China decided to embrace harm reduction – in the form of methadone maintenance – the numbers of clinics and attendees increased drastically [40].

Tijuana is located at the Mexico-US border, a fluid and liminal boundary through which people, media broadcasts, new coverage, and policies flow towards the north and south. Unsurprisingly, the policy environment of Tijuana may be as affected by harm reduction policy approaches from the United States – specifically San Diego – as it is by the policy approaches of the Mexican Government. The central harm reduction approach in the United States is methadone-based drug treatment and state-operated or privately run NEPs. An illegal needle exchange program was implemented and operated in San Diego for many years prior to the implementation of a legal NEP in 2000 [41], at which time NEPs were legalized in 2000 in California if a local health emergency was declared. San Diego declared a health emergency in 2000, and in 2002, San Diego implemented a legal pilot NEP [42] that is operating today. Significant media broadcasts of the implementation of NEP in San Diego occurred in both the English-language and Spanish-language news media. Thus, the perception of stakeholders in Tijuana, who were surely aware of the barriers that NEP implementation had faced in San Diego, may have been influenced by the policy of the United States. This possibility is reflected in our results, with 75% of the respondents finding NEP to be the most acceptable, and over half finding NEP to be the most feasible. The harm reduction interventions that are not implemented in the United States (SIFs and vending machines) were seen as less acceptable and less feasible.

Additional studies have stressed the importance of not simply creating policy, but also closely observing its implementation to assure it is having the intended affect. For example, while Australia has extensive policy commitments to harm reduction, studies have shown that in some locations policing practices exert a powerful influence on IDU risk behavior, resulting in a reported fear of carrying needles or attending NEPs [43,44]. Though many of the respondents listed culture as a barrier to harm reduction, previous studies have critiqued this act of listing culture as a barrier and instead stressed the importance of integrating systems of local knowledge into interventions [45]. For example, studies targeting malaria have found it important to first generate a list of local terms associated with malarial symptoms, as often times the translation and western description of "malaria" does not match indigenous cultural understandings [46]. Other studies have stressed the importance of understanding culture as a fluid and malleable entity that both affects people and is affected and changed by them [47,48]. Further exploring local systems of meanings, symbols, and indigenous health knowledge will allow interventions to be more applicable and integrated into cultural understandings [49,50,48]. For example, persuading a church to host a NEP or distribute condoms among its parish may be a powerful symbolic approach that mediates the perception that "religion" is a barrier, In this way, culture can both be acknowledged and integrated into existing program to serve as a benefit as opposed to a barrier.

This study has important limitations. Interviews were conducted with diverse participants across various sectors; participants represented both policy and decision makers and those who interacted daily with IDUs. While we built a diverse sample, we could only speak with those who consented to be interviewed – and some sectors were missing. For example, we were not able to interview high-level officers in the police department as they refused our interview requests. These results are not generalizable to stakeholders in other cities in Mexico, as our study by definition explored local perceptions; perceptions likely influenced by the geographic position of Tijuana as a border city and by its location as an important way-station on a drug trafficking route. We did not use a theoretical sampling framework, but we reached a saturation of the key themes, providing confidence in our results.

One factor that was both a limitation and an important finding of this study was that some of the interviewees had not previously heard of the three harm reduction interventions, making it potentially difficult to form a complete opinion after hearing a brief description. Our results suggest that harm reduction interventions are needed in Tijuana and that some stakeholders believe it crucial to increase awareness and understanding prior to implementation. In order to raise awareness, there must be a facilitation of intersectoral collaboration and discussion between stakeholders, and careful acknowledgement of the socio-cultural factors specific to Tijuana in order to increase the possibilities of implementation. As these suggested changes are implemented, the NEP in Tijuana will continue providing sterile injection equipment in order to slow the spread of blood-borne diseases among injection drug users, and serve as a successful example for future interventions throughout Mexico.

## Competing interests

The authors declare that they have no competing interests.

## Authors' contributions

MMP contributed to the data collection, analysis and drafting of the manuscript. RL and AM aided in the collection and analysis of interview data. CAL participated in the design of the study and all authors read and approved the final manuscript. SAS conceived of the study, participated in its design and coordination, and helped with the drafting and editing of the manuscript. PC contributed to the conception, theory, and design of the study, and aided substantially in the development of the manuscript. MLZ helped with the coding of the data and the development of the manuscript. CMR contributed to the development and design of the study, provided advice on key stakeholders who should be contacted and offered technical support.
